# Online News Coverage of the Sugar-Sweetened Beverages Tax in Malaysia: Content Analysis

**DOI:** 10.2196/24523

**Published:** 2021-08-18

**Authors:** Muhammad Faiz Mohd Hanim, Budi Aslinie Md Sabri, Norashikin Yusof

**Affiliations:** 1 Centre of Population Oral Health and Clinical Prevention Studies Faculty of Dentistry Universiti Teknologi MARA (UiTM) Sungai Buloh Malaysia

**Keywords:** sugar-sweetened beverages, obesity, taxes, media content analysis, public health policy, media content, public health, netnography, malaysia, budget

## Abstract

**Background:**

In Malaysia, the Sugar-Sweetened Beverages (SSBs) tax was announced during the parliament's 2019 Budget Speech. The tax was slated to be enforced by April 2019 but was later postponed to July 2019. The announcement has since generated significant media coverage and public feedback.

**Objective:**

This study presents a qualitative and quantitative cross-sectional study using netnography to examine how Malaysian online news articles responded to the SSBs tax after the announcement and postimplementation.

**Methods:**

Online news articles published on popular online news platforms from November 2018 to August 2019 were downloaded using NCapture and imported into NVivo for analysis using the inductive approach and thematic content analysis following the initial SSBs implementation announcement.

**Results:**

A total of 62 news articles were analyzed. Most of the articles positively portrayed the SSBs tax (46.8%) and highlighted its health impacts (76%). There were 7 key framing arguments identified in the articles. The positive arguments revolved around incentivizing manufacturers to introduce healthier products voluntarily, positive health consequences, the tax’s impact on government revenue, and the use of the generated revenue toward beneficial social programs. The opposing arguments included increased operating costs to the manufacturer, the increased retail price of drinks, and how the SSBs tax is not a robust solution to obesity. The top priority sector considered in introducing the tax was the health perspective, followed by economic purposes and creating policies such as regulating the food and drinks industry.

**Conclusions:**

The majority of online news articles positively reported the implementation of the SSBs tax in Malaysia. This suggests media played a role in garnering support for the health policy. As such, relevant bodies can use negative findings to anticipate and reframe counteracting arguments opposing the SSBs tax.

## Introduction

Most noncommunicable diseases (NCD) such as cancer, cardiovascular diseases, dental caries, and diabetes share a common risk factor that can be attributed to high BMI and obesity [1-5]. Previously, NCDs were primarily a problem in high-income countries but have become prevalent in low-income and middle-income countries [6]. One of the main factors contributing to the rising prevalence of NCDs is the increased consumption of sugar-sweetened beverages (SSBs) [6]. The amount of sugar added to SSBs is quite substantial, and a 330 ml or 12 oz portion of a sugar-sweetened carbonated drink usually contains about 35 grams of sugar, which is equivalent to almost 9 teaspoons of sugar, offering little nutritional value [7].

The widespread availability of SSBs and their convenience have led to increased consumption worldwide [8,9]. Globally, consumption of SSBs is highest among young adults aged between 20-39 years [9]. Studies from Han [8] and Hazam [10] found that 40% to 70% of young adults in low-income countries consumed SSBs daily, especially in the form of energy drinks and soda or soft drinks.

One way to curb increased SSBs consumption is to introduce a tax or excise duty [11]. Most policymakers view this tax as one of the tools to tackle the obesity epidemic by limiting the choices available to consumers, encouraging manufacturers to limit the sugar content of their products, and creating a revenue stream to combat health-related problems caused by the SSBs [11,12].

Several countries have introduced SSBs taxation, but its implementation has received mixed reactions. Some groups oppose it, with health advocates who lobbied for SSBs taxation supporting it [11-14]. Opponents of SSBs taxation question its effectiveness in addressing the obesity problem and do not believe SSBs taxes alone can tackle obesity and overweight problems [13-16]. Nonetheless, SSBs taxation is gaining momentum because of its relative ease of implementation compared to other food or nutrition policy options [17]. Even though the United States has not implemented a nationwide tax, there have been some successes among several local and city governments that have adopted a similar tax [13]. For example, in Berkeley, California, the sugar tax resulted in a 21% decrease in soft drink consumption among the low-income neighborhoods [13,18]. While in Philadelphia, Pennsylvania, the SSBs consumption fell by 26% 2 months after the implementation of a beverage excise tax [18]. In the United Kingdom, a 3-tiered levy on SSBs was implemented in April 2018, in which the amount of levy imposed depends on the sugar content per 100 ml [19]. This has led to soft drink manufacturers reducing the sugar content of their products and a significant decrease (approximately 50%) in sales of soft drinks subjected to the levy [19].

In Australia, the final report by the “Select Committee into the Obesity Epidemic in Australia” recommended that the Australian government introduce a tax on SSBs with the objectives of reducing consumption and accelerating the reformulation of products [14]. Despite this recommendation, there was opposition, especially from the beverage industry, and there was even a consideration to reject its implementation. However, in 2018, the Australian Beverages Council announced it would seek to reduce the sugar content in beverages by 10% by 2020 and a further 10% by 2025 [13].

In Malaysia, the SSBs tax proposal levied 40 cents per 1 liter for beverages containing more than 5 grams of sugar per 100 ml and fruit juices with 12 grams of sugar per 100 ml. The tax was scheduled to be enforced in July 2019 [20].

Research has suggested that the media can influence public perception and the potential acceptance of public health policies [21-23]. Mass print or online media provide a comprehensive platform to reach out to the public, and they can effectively put health issues on the public agenda and determine how these issues are framed [23]. This framing process involves emphasizing some aspects of a debate while downplaying others. Framing can include representations of the severity of societal problems, their causes, and the potential effectiveness of proposed solutions. Thus, framing can influence the audience's perception and evaluation of different approaches to address public health problems [23]. Media framing of the SSBs tax within the context of sugar and SSBs consumption issues could influence the public acceptance of this upstream solution via taxation.

Policy articles posted on online media platforms play an important role in identifying supporting and opposing messages, and the challenges stakeholders may face when advocating for policy agendas such as SSBs taxation and tobacco control. A range of studies on the content analyses of media coverage on SSBs taxes and tobacco-related taxes have been published [21-28]. The strategic messaging by proponents of SSBs taxes can influence public opinion and shape policy development [22,25]. In a study conducted by Niederdeppe, Jeff et al [26] examined the news coverage of public debates on SSBs to illuminate how the news media frames these debates. Most of the debates framed the issue in favorable ways [26]. For example, the comprehensive media coverage generated by the Australian government’s announcement of legislation mandating that tobacco products be sold in plain packaging provided an opportunity for tobacco control advocates to anticipate and counteract arguments opposed to the legislation [28]. Likewise, the media coverage of sugar-based taxes and SSBs consumption has helped shape policy to favor fiscal solutions that curb SSBs consumption and drive greater public acceptance of the sugar levy in the United Kingdom [22].

In Malaysia, many articles regarding the SSBs tax have been written in online news media. Media coverage of any particular issue is generally linked to the agenda or priorities of the government in power. However, during our study period, the media, especially the newspapers, were not linked to either government or the opposition, as reported in the Star Online, April 18, 2019. The article stated, “Malaysia has jumped 22 places to 123^rd^ in the latest World Press Freedom Index, compiled by Reporters Without Borders (RSF),” becoming a top-ranked country among other ASEAN (Association of Southeast Asian Nations) countries [29]. In 2020, Malaysia's rank (101/180, showed that the Malaysian media were still enjoying their freedom. This showed that the articles written by the media are less likely to be biased but rather featured the media’s accurate sentiment as reported through their online platforms [29]. Furthermore, the content of Malaysian-based online news platforms typically mirrors their paper or print-based counterparts, allowing for the generalizability of online news content analyses to the general media.

Since its announcement, no study has been conducted to examine the online news articles' responses regarding Malaysia’s SSBs tax. Thus, this study examines the response of Malaysian online news articles and how the media framed the arguments during the 10 months after its announcement and up to its implementation.

We also documented any sector-related and health-related issues used to justify the SSBs tax in the news reporting. All news outlets were captured to build a picture of which key framing arguments and messages were featured in the news articles.

## Methods

### Collection of Data

According to the reporting website, Malaysia's total number of online news platforms varies from 29 to 35 platforms [30,31]. In this study, the top 10 Malaysia-based online news platforms with total unique visitors in July 2019 (the latest information available) reported by the Malaysian Digital Association were purposefully selected to ensure a sample coverage representing various Malaysian online news readers [32]. As a result, the top Malaysia-based online news platform visited was a Malay version of the Harian Metro (Metro Daily), with 4.2 million unique visitors. Conversely, the online news platform with the lowest unique visitor rates included in the sample was the English newspaper version of The New Straits Time, which recorded 2.2 million unique visitors [32].

Data collection included news articles written between November 2018 to August 2019. Separate searches were conducted in the respective online news platforms. Suggestive keywords used were “sugar tax,” “sugar-sweetened beverages tax,” and “soda tax.” Suggestive keywords in other languages such as Malay, Mandarin, and Tamil were translated using Google translate. The news articles that appeared in the search results were scanned for relevance and downloaded using either the web browser extension NCapture (version 1.0.290.0; QSR International) and then imported into NVivo (version 12, QSR International). The articles were read, and to avoid duplication, the articles were excluded if the Malaysian online news platform did not produce the original articles.

### Content Analysis

All news articles were read in full, and 2 researchers conducted the coding. The coding variables are shown in Textbox 1. After the researchers separately coded the articles, the results were discussed and calibrated until a consensus was reached. News articles were coded and analyzed for the topic, framing arguments, overall slant, related sectors, health-related issues used to justify the SSBs tax, and direct quotes or position statements. Coding categories were developed iteratively, adapting a methodological approach similar to the one taken by Christina Watts and Becky Freeman [21]. Each article was coded differently, representing the primary message of the article.

**The coding variables used to code the arguments in the articles.**

- **Topic:** Overall, what is the news article about? (1 topic per article coded).
- **Framing argument:** What is the argument presented concerning the introduction of the sugar-sweetened beverages (SSBs) tax in Malaysia? The framing argument was determined by identifying the argument presented most frequently within the articles.
- **Slant:** Is the article presenting the SSBs tax as a positive or negative policy? Or is the article neutral toward the tax? A positive slant is defined by a framing argument that favors SSBs taxation, and a negative slant is defined by a framing argument that is opposed to SSBs taxation. A neutral slant is defined by the absence of a framing argument and the presentation of a neutral debate.
- **Related sectors:** Which related sectors were mentioned or highlighted in the article as justification for SSBs taxation?
- **Health-related issues:** What type of health-related issues used as justification for SSBs taxation were mentioned or highlighted in the article?
- **Direct quotes or position statements:** Who is quoted or paraphrased in the article with a position or opinion on the SSBs tax?

Articles were also content analyzed and coded according to the most frequently presented argument in the article. Each framing argument was categorized as either in favor of, against, or neutral toward the SSBs tax. Articles were also analyzed for the justification used in introducing the tax with references to related sectors, health-related issues, and direct quotes or position statements about implementing the SSBs tax.

## Results

### Overview

Out of the 79 online news articles, 17 (21.5%) were excluded due to duplications (n=4, 23.5%), readers' letters, editors' opinions, and infographics (n=13, 76.5%). The final content analysis consisted of 62 news articles.

### Topics and Overall Slant

The topics and overall slants are summarized in Table 1. The most frequently highlighted topic concerned the impact of SSBs taxation on people’s health (40.3%), followed by the implementation announcement (21%), and the contribution of the SSBs tax toward government revenue (6.5%). There was an overall positive slant towards SSBs taxation (46.8%), primarily highlighting positive health outcomes as the main impact of the SSBs tax (Table 1). On the other hand, there was a 16.1% negative slant, which includes the manufacturers' response towards the SSBs tax (33.3%) and its impact on the consumers (50%). However, online newspaper articles were neutral when reporting the SSBs tax announcement in Malaysia.

**Table 1:** Article themes by overall slant.

Topic	Brief description	Slant, n (%)			
		Positive	Negative	Neutral	Total
Announcement or implementation of the SSBs^a^ tax	Articles report on the announcement or implementation of the SSBs tax	0 (0.0)	0 (0.0)	13 (100)	13 (100)
Health effects	Articles explore the impact of SSBs taxation on health	19 (76)	2 (8)	4 (16)	25 (100)
Government revenue	Taxation on SSBs will generate another source of income for the government to reduce the country’s deficit	2 (50)	0 (0.0)	2 (50)	4 (100)
Manufacturer response	Articles report on how the food industry or beverage companies react to SSBs taxation	7 (58.4)	4 (33.3)	1 (8.3)	12 (100)
Consumer response	Articles report on the impact of the SSBs tax on the consumers	1 (12.5)	4 (50)	3 (37.5)	8 (100)
Total	N/A^b^	29 (46.8)	10 (16.1)	23 (37.1)	62 (100)

^a^SSBs: sugar-sweetened beverages.

^b^N/A: not applicable.

### Framing Arguments

There were 7 key framing arguments used in the online news articles (Table 2). Framing arguments primarily supported taxation (46.8%), with 16.1% of the arguments opposing the taxation, and 37.1% framed as balanced arguments as they did not have any primary framing arguments. The majority of arguments supporting SSBs taxation reported the positive health gains of reducing SSBs consumption in the general public (21.7%). Some examples include nongovernmental organizations (NGOs) who applauded the SSBs tax because it will encourage healthy lifestyle behaviors and help reduce the incidence of NCDs (eg, diabetes) in the country [25,26].

The articles also argued that the tax incentivizes SSBs manufacturers to reduce the sugar content and introduce healthier products (14.5%). About 8% of the arguments reported that the tax would help to increase the government's revenues, and the extra resources collected could be used to treat diseases that list sugar consumption as one of the risk factors. It was also noted that the extra resources could also provide a free, nutritious, and healthy breakfast to the school children. Articles that presented arguments opposing the SSBs tax argued the tax would increase manufacturers’ operating costs due to reformulating their products, resulting in profit reduction (8.1%). Approximately 4.8% of online articles reported the tax was not an appropriate solution for diabetes control. However, only 3.2% opposed SSBs taxation due to the perception that the price for all the drinks will increase. An example of a statement regarding the price hike included “implementation of excise duty on sugar-sweetened beverages today has resulted in a price increase for most of the products between RM0.20 to RM0.70… few sundry shops have already adhered to the price adjustment of their goods…the products affected are carbonated drinks, ready-to-drink coffee, milk, juices, and canned and packet drinks” [33].

**Table 2:** Framing arguments presented in online news articles.

Framing argument		Articles, n (%)
**Argument supporting the SSBs^a^ tax**		29 (46.8)
	Incentive for the manufacturer to introduce a healthier product voluntarily	9 (14.5)
	The collection of the SSBs taxes will increase the government revenue and help to reduce the burden of future treatment costs in public healthcare facilities	5 (8.1)
	Positive health consequences of reducing SSB consumption	13 (21.7)
	Revenue generated will be used to help children (eg, providing free breakfasts for all school children)	2 (3.2)
**Argument opposing the SSBs Tax**		10 (16.1)
	SSBs taxation will increase the operating costs of reformulating products to avoid taxation, resulting in decreased profit margins	5 (8.1)
	SSBs taxation is not the solution to obesity	3 (4.8)
	The tax might increase the overall price as the SSBs will be taxed twice (SST and SSBs Tax)	2 (3.2)
Balanced argument		23 (37.1)

^a^SSBs: sugar-sweetened beverages.

### Sector-Related Purposes

Online news articles mentioned a few sectors used to justify the introduction of the SSBs tax. The top priority sector used as consideration for the tax was presented from the health perspective (35.5%), followed by economic purposes and creating healthy policies (22.6%), and regulating the food and drinks industry or manufacturers (9.7%). However, few sectors, including health, were mentioned in the online news articles discussing the implementation of the tax (9.7%).

### Health-Related Issues

The primary consideration for introducing the SSBs taxation was health-related issues. The main highlight for this argument was that sugar is a high-risk factor for diabetes (21%), followed by general health (42.9%) and obesity (10.7%). However, there was no mention of caries or dental problems as one of the reasons justifying SSBs taxation.

### Quotes or Position Statement

Most of the quotes or position statements were from the manufacturers (17.7%). The main concern of the beverage industry was the increased cost that they had to bear once the tax is imposed. The finance ministry, which made up 16.1% of the quotes, was concerned about how the tax would be implemented, while the health ministry hoped that the tax would lead to reduced consumption of SSBs by consumers and improved health gains (14.5%). However, 1 NGO argued that the tax would not solve any health-related issues. Instead, it will have a negative impact on the lower-income populations [34] due to consumers incurring the additional cost imposed by the SSBs tax. In contrast, 7 out of 8 NGOs agreed SSBs taxation could alleviate the government’s financial burden in the long term as fewer expenses may be required to treat sugar-related diseases [35].

Online news articles also quoted statements from other government stakeholders such as the Director-General of Royal Malaysia Customs Department (8.1%), the Deputy Prime Minister (6.5%), the WHO (World Health Organization; 6.5%), economic analysts from local institutions (3.2%), political parties (1.6%), and the Ministry of Domestic Trade and Consumer Affairs (1.6%). The remaining articles either quoted more than 1 organization (6.5%) or did not quote any organization (4.8%).

## Discussion

### Principal Findings

This study aimed to explore and generate an in-depth understanding of the media response to the SSBs taxation policy. Most of the online news articles were positive towards the implementation of SSBs taxation. They primarily addressed the benefits of SSBs taxation to the general society and the individual by advocating that reduced consumption of SSBs will improve one's overall health by reducing the risk of sugar-related diseases [36-38]. According to Vermeen et al [39], an additional 20% tax on SSBs would result in a modest reduction in BMI, translating into positive health gains adding up to approximately 170,000 healthy life years over the lifetime of the Australian adult population [39].

The Malaysian government also hoped that the SSBs manufacturers could reformulate or reinvent their products to make healthier beverages without imposing any policy. The suggestion from the finance minister stated that manufacturers need to lower the sugar content to avoid the imposed SSBs tax [37,38,40-45]. Out of the 62 articles analyzed, 29 (46.8%) articles supported the taxation. This is a positive result for public health advocates, indicating that the online news articles on the SSBs tax were generally accepted as reputable, newsworthy, and fundamental to reducing the overconsumption of sugar.

The articles also highlighted that taxation would increase government revenue and reduce the burden of future treatment costs on public healthcare facilities, which are heavily subsidized by the government [36,38]. Another finding supported the SSBs tax as it can reduce the disease burden and health care costs associated with sugar consumption [39].

The articles also emphasized using the tax revenue to benefit the school children by providing them with healthy and nutritional breakfasts. The articles aimed to garner public support, especially from the parents, as the SSBs tax would benefit their children rather than cause harm [46]. This is also in line with the Sustainable Development Goal 2 “to ensure that every child, young person, and woman received a nutritious, safe, affordable and sustainable diet that they need to reach their full potential” [47]. It is crucial for children to grow and learn and participate in their communities during their school learning days. The MyBreakfast study on breakfast consumption among Malaysian primary and secondary children highlighted that 1 out of 4 children skipped breakfast, concluding a regular breakfast is associated with healthier body weight and should be encouraged [48].

However, despite the positive slant of most online news articles, articles regarding the manufacturer and consumer response contributed to the negative slant. There is a possibility that these 2 groups were the most affected by the SSBs tax. One of the manufacturers' concerns was the increase in operating costs to reformulate their products, resulting in a price hike on SSBs that is passed to the consumers [49,50]. Another respondent stated that the SSBs tax is not a solution to diabetes and associated the ineffectiveness of SSBs taxation with the tobacco tax, which did not solve society's smoking addiction [51]. A systematic review by Escobar et al [52] suggested that an increase in the price of SSBs was associated with a decrease in consumption. The higher the price increase, the more significant the reduction in consumption. However, the argument against the imposition of the SSBs tax is that it is regressive and could negatively impact lower-income households who spent a considerable portion of their income on inexpensive, prepackaged consumable goods compared to the higher-income households [52]. However, a study by Bourke and Veerman [53] in Indonesia suggested that while an excise tax on SSBs could decrease the incidence of NCDs in all groups, the health benefits will accrue primarily in the high-income groups as they consume more sugary drinks and pay more of the tax than the lower-income group and thus the tax is not regressive [53].

Most of the articles portrayed potential health outcomes as the government's justification in implementing the SSBs tax. Evidence supports that such taxes could substantially reduce consumption and reduce the incidence of diabetes and obesity [6,52]. It is also well-known that excessive sugar intake is a significant risk factor for caries development. Findings from the latest Malaysian oral health surveys showed that even though caries prevalence has reduced over the years, it needs to be addressed as it is still high among certain age groups, particularly children aged 6 years (71.3% in the 2015 National Oral Health Survey of Preschool Children) and adults (88.9% in the 2010 National Oral Health Survey of Adults) [54]. Jevdjevic et al [24] showed that SSBs taxation might reduce the caries-related burden and improve oral health, especially among the younger age groups [24].

Given the high media reliance on politicians and key decision-makers to portray social problems, there is a potential for widespread public acceptance or rejection of SSBs taxation [55]. Quoting the appropriate spokesperson to avoid inaccuracies in news media articles regarding SSBs taxation is crucial [55]. A study by Bødker et al [56] demonstrated that active industry lobbying and consequent judicial actions could undermine policy support from all stakeholders [56]. Another study found that SSBs manufacturers did have substantial coverage in the Malaysian media, allowing them to express their perspective on the tax. The issues they put forward mainly involved the impact of taxation on their businesses and the consumers. They attempted to portray that the taxation will increase their operation costs, which will ultimately result in higher costs for the consumers due to the increased retail price of SSBs [57,58]. This perspective may result in public outcry, which will negatively impact policy and ultimately undermine public health efforts to reduce SSBs consumption.

This study analyzed a current debate in the media that supported reducing the obesity and diabetes prevalence by introducing the SSBs tax as a sugar intake reduction policy. The findings have helped to frame evidentiary public health policy more broadly. The total number of articles written regarding SSBs taxation showed how public health studies and facts could promote media agenda setting. Public health proponents will welcome the substantial portrayal of sugar and SSBs as a social health issue, primarily driven by the food and drink industries and best tackled by policy measures. Moreover, while the framing arguments produced by online news outlets were overwhelmingly favorable to public health advocacy and supporting coverage outnumbered opposition coverage, public health advocates should be mindful of the predominance of opposition outlets around the SSBs tax announcement. A concerted media advocacy campaign could have mitigated the wave of SSBs taxation opposition.

The findings of this study are significant because readers’ comments and public views on online news coverage can also influence decisions about the final form of the SSBs tax, which may contribute to its successful implementation and overall efficacy. Thus, public health advocates should understand and study public opinion to ensure the effective implementation of the levy [16].

### Study Limitations

It is also important to note that the findings from this study are subject to a range of limitations. For example, news articles were not analyzed for readership numbers or the number of times each article was shared on social media; therefore, the articles' overall reach could not be determined. Another limitation of the content analysis was that the coding was based on the subjective assessment of the 2 independent researchers.

### Conclusions

The findings showed that most online news articles were written with a favorable slant towards implementing the SSBs tax. This suggests the media played an important role in supporting the health policy. Besides, the policymakers should also complement the SSBs tax with other strategies such as adequate health education and promotion as there may be a lack of awareness in the general public. Hence, the potential benefit of the SSBs tax will go beyond merely influencing price-based purchasing behavior and extend to the normalization of sugar-free beverage consumption, including plain drinking water. Furthermore, the opposing arguments towards the SSBs tax found through this study could be used by relevant bodies to anticipate opposition and assist in reframing and counteracting arguments opposed to the SSBs tax.

## Abbreviations

SSBs: sugar-sweetened beverages
NCD: noncommunicable disease
NGO: nongovernmental organization
